# Intertwining Threshold Settings, Biological Data and Database Knowledge to Optimize the Selection of Differentially Expressed Genes from Microarray

**DOI:** 10.1371/journal.pone.0013518

**Published:** 2010-10-20

**Authors:** Paul Chuchana, Philippe Holzmuller, Frederic Vezilier, David Berthier, Isabelle Chantal, Dany Severac, Jean Loup Lemesre, Gerard Cuny, Philippe Nirdé, Bruno Bucheton

**Affiliations:** 1 INSERM, Unité 844 - Montpellier, France; 2 Unité Mixte de Recherche IRD - Centre de CIRAD 177- Montpellier, France; 3 Montpellier GenomiX; c/o Institut de Génomique Fonctionnelle – Montpellier, France; 4 Institut de Recherche en Cancérologie de Montpellier – INSERM, Unité 896, Université Montpellier1 - Montpellier, France; Georgia Institute of Technology, United States of America

## Abstract

**Background:**

Many tools used to analyze microarrays in different conditions have been described. However, the integration of deregulated genes within coherent metabolic pathways is lacking. Currently no objective selection criterion based on biological functions exists to determine a threshold demonstrating that a gene is indeed differentially expressed.

**Methodology/Principal Findings:**

To improve transcriptomic analysis of microarrays, we propose a new statistical approach that takes into account biological parameters. We present an iterative method to optimise the selection of differentially expressed genes in two experimental conditions. The stringency level of gene selection was associated simultaneously with the p-value of expression variation and the occurrence rate parameter associated with the percentage of donors whose transcriptomic profile is similar. Our method intertwines stringency level settings, biological data and a knowledge database to highlight molecular interactions using networks and pathways. Analysis performed during iterations helped us to select the optimal threshold required for the most pertinent selection of differentially expressed genes.

**Conclusions/Significance:**

We have applied this approach to the well documented mechanism of human macrophage response to lipopolysaccharide stimulation. We thus verified that our method was able to determine with the highest degree of accuracy the best threshold for selecting genes that are truly differentially expressed.

## Introduction

Microarray technology [1] has emerged in the last decade as the favoured method for large-scale gene expression studies. The technique can be used to simultaneously analyse the expression of thousands of genes and requires relatively small amounts of starting RNA material, therefore it provides a powerful tool for the comprehensive analysis of tissue or cell biology in response to a given stimulus such as; an infection [2], [3], a disease such as cancer [4]–[6], chemoresistance [7] or development, e.g. cell differentiation [8]. This means that the relationships between genes and their involvement in specific cellular functions can be better characterized. However, owing to the large number of genes and to the small number of samples, there are many statistical problems associated with microarray data [9], [10], which makes the detection of differential gene expression a challenging task. One of the main problems is the huge amount of data generated by microarray technology. Consequently, algorithms such as Ingenuity Pathway Analysis, LSGraph, Cognia Molecular, Metacore, or Bibliosphere were developed to analyse and understand complex biological systems. However, distinguishing genes that undergo expression variation (EV) among all the genes analysed remains difficult. Consequently, the normalization of gene expression data [11] and the development of methods to identify genes undergoing expression variation (EV) would represent an important step forward. A number of papers have described methods for assessing selected dataset requirements in microarray experiments using statistical criteria [12]. However, in all cases, the selection of genes undergoing expression variation is associated with a stringency parameter. Lee and Whitmore [13] used an ANOVA model and provided power calculations for various alternative models. Muller et al. [14] used a decision-theoretic approach and a hierarchical Bayes model. Wei et al. [15] examined the roles of technical and biological variability, in determining a selected data set. Pawitan et al. [16] assumed that genes are independent and have equal variance, and the paper reports on false discovery rates and sensitivities. Sample size calculations for a microarray experiment package (*ssize.fdr* package) [17] also assumed that the genes are independent, but pilot data is used to estimate the variance. It focused on test power and Type 1 errors (false negatives).

Increasing the stringency levels leads to the selection of genes displaying the largest expression differences and thus to an increase in Type 1 error risk. However, the lowering of the stringency levels of selection means genes with a lower level of expression variation are also chosen. Unfortunately, it also leads to an increase in the risk of Type 2 errors (false positives). Consequently, choosing the appropriate stringency threshold is of crucial importance.

In this paper we address these issues, and propose a new methodology for the analysis of micro-array transcriptional data in which the stringency analysis threshold is not only determined using statistical approaches but also intertwined with biological considerations to allow for a more specific and sensitive selection of the differentially regulated genes.

In our work, we statistically link gene selection stringency to an expression variation or its p-value. Thereafter, the occurrence rate parameter is associated with the percentage of donors whose transcriptomic profile is similar. Next, we associated gene selection and occurrence rate in order to further refine gene selection. Finally, knowledge of biological interactions, canonical pathways and these differentially expressed genes are then intertwined to obtain an accurate threshold.

In order to validate this new statistical approach, we applied this methodology to a well-known cellular activation model, i.e. the LPS activated human peripheral blood derived macrophages [18]–[20]. For study purposes, Monocyte Derived Macrophages (MDM) from 6 blood donors were stimulated, or not, using LPS. As the macrophage response to LPS has been extensively studied (about 8700 articles and 300 reviews). This gave us the framework with which we could monitor the evolution of different analysis parameters in order to maximize those providing the most useful information. We clearly observed that an analysis with an occurrence rate of 100% gives the most significant results and enables the detection of genes with low expression variation differences. However, there is the inherent risk of missing important genes involved in the macrophage response to LPS. On the one hand, increasing the occurrence rate reduces the number of genes selected, but increases the risk of missing relevant genes (Type I error). On the other hand, decreasing occurrence rate will, of course, increase the number of genes selected, but also the risk of “noise” i.e. irrelevant genes that would pollute the selected dataset (Type II error). This would result in the inclusion of non-relevant genes for macrophage response to LPS. Our analysis clearly showed that information in the dataset increased until an occurrence rate of 4/6 whereas this information was partially lost for occurrences<4/6 because of increased noise within the data set.

We clearly demonstrated that, when compared to other existing methods, our statistical approach selects differentially expressed genes with the highest degree of accuracy. It does so by providing the most sensitive and specific threshold for gene selection.

## Results

### Selection of intertwined EVs and occurrence thresholds for the analysis of gene expression

In order to identify macrophage genes which expression varies during LPS activation, total RNA was harvested from human monocyte-derived macrophages of the 6 donors cultured with, or without, LPS activation. EV normalisations were robust and provided accurate EV values.

#### Stringency level setting as a key parameter for gene selection monitoring

Microarray data were analysed using the EV method in order to identify differentially regulated genes on paired data from the same donor. Analysis of gene expression variation was carried out using two different comparison approaches. Using a standard approach we selected genes for which the EV mean measurement for the 6 donors was associated with a p value≤0.01. We obtained an EV of 2.32, and were able to identify 68 or 69 differentially expressed genes when using the mean or the median, respectively.

In the second approach, we used a higher p-value (p≤0.1 corresponding to a value of EV≥1.28). In this case, analysis was performed with a decreasing EV occurrence from N = 6/6 to N≥3/6 (Table 1). The probability that a gene will be differentially expressed is a function of individual probabilities. Therefore, if a gene has an EV≥1.28 in at least 4 out of the 6 individuals (EV occurrence≥4/6), its minimum p-value is 10^−2^. For genes with an EV occurrence = 6/6 a minimum p-value of 10^−6^ can be reached. At this last occurrence rate, 114 genes were selected and the number of selected genes increased to 189 (N≥5/6), 300 (N≥4/6), and 461 (N≥3/6) for lower EV occurrences (Table 1).

**Table 1:** Evolution of common gene output according to EV occurence.

Stringency	Genes output	Eligible network	Common genes	Common genes %	Genes in Main network	Specific genes in main network	Genes % in main network	Best score of main network
= 6/6	114	79	8	13.13	76	68	86.08	49
≥5/6	189	131	18	13.74	125	107	81.68	45
≥4/6	300	202	63	31.19	193	130	64.36	38
≥3/6	461	258	49	18.99	246	197	76.36	43

Each possible EV occurrence stringency level was tested, i.e. from  = 6/6 down to ≥3/6. EV analysis provides “Gene output” in the second column. Using Ingenuity Pathway Analysis software we obtained the following data. The “Eligible Network” column which gives the number of genes belonging to a network. The “Common Genes” column represents the total number of genes which have been found in at least two different networks. The “Common gene %” column provides the percentage of genes shared by different networks. The next column gives the total number of genes in the main network, from which it is possible to calculate the number of specific genes (genes in the main network minus genes common to at least two networks). The second last column presents specific genes as the percentage of all genes in the main network (column Gene % in main network). Finally, the table displays the “best score of the main network” for each stringency level.

#### Intertwining threshold settings and EV occurrence rates

IPA analyses were then carried out on the different sets of selected genes to determine the most suitable EV occurrence threshold for identifying the largest set of genes associated with LPS activation. As expected, in the case of decreased statistical stringency, the following parameters; the number of IPA mapped genes, the number of network eligible genes, and the total number of identified networks, increased steadily with decreasing EV occurrence (Table 1). Therefore, these parameters are not appropriate when determining the appropriate EV occurrence threshold. However other analysis parameters did show interesting features. The number of genes common to the different identified networks increased to a maximum of 63 for an EV occurrence≥4/6 and dropped to 49 for an EV occurrence≥3/6. The ratio of genes connecting networks according to the total number of genes associated to networks was twice as high (31.19%) for an EV occurrence≥4/6 when compared the other occurrences tested. This finding demonstrates the optimal structuration of genes selected at the above threshold.

This structure forming effect is lost for lower EV occurrence as newly added genes tend to fall into new networks unlinked to the ones identified at higher EV occurrence. Indeed, the distribution of the genes from the best network (network 1) for an EV occurrence = 6/6 are mainly found (56.5%) in the first network, which has an EV occurrence rate of ≥5/6 (Table 2). Similarly, 70.8% of genes in the best network with an occurrence rate of ≥5/6 are found in the first network with an occurrence rate≥4/6. However, the genes in the first network with an occurrence rate≥4/6 are not found in the first network at an occurrence rate of ≥3/6, but are mainly found in the fourth network.

**Table 2:** Percentages of genes shared between networks for consecutively paired stringency levels.

N≥5/6N = 6/6	Network 1	Network 2	Network 3	Network 4	Network 5
Network 1	**56.5%**	43.5%		8.7%	4.3%

N≥4/6N≥5/6	Network 1	Network 2	Network 3	Network 4	Network 5
Network 1	**70.8%**	42.0%		12.5%	8.3%

N≥3/6N≥4/6	Network 1	Network 2	Network 3	Network 4	Network 5
Network 1		11.5%	15.4%	**50.0%**	3.8%

Best score are in bold case.

Best scores are in bold print. As long as the best scores are shared between paired N°1 networks, the stringency level may be too high, and should therefore be lowered. Once the best score is no longer shared between N°1 networks, the stringency level is deemed too low. Therefore, the preceding stringency level should be selected.

**Figure 1:**
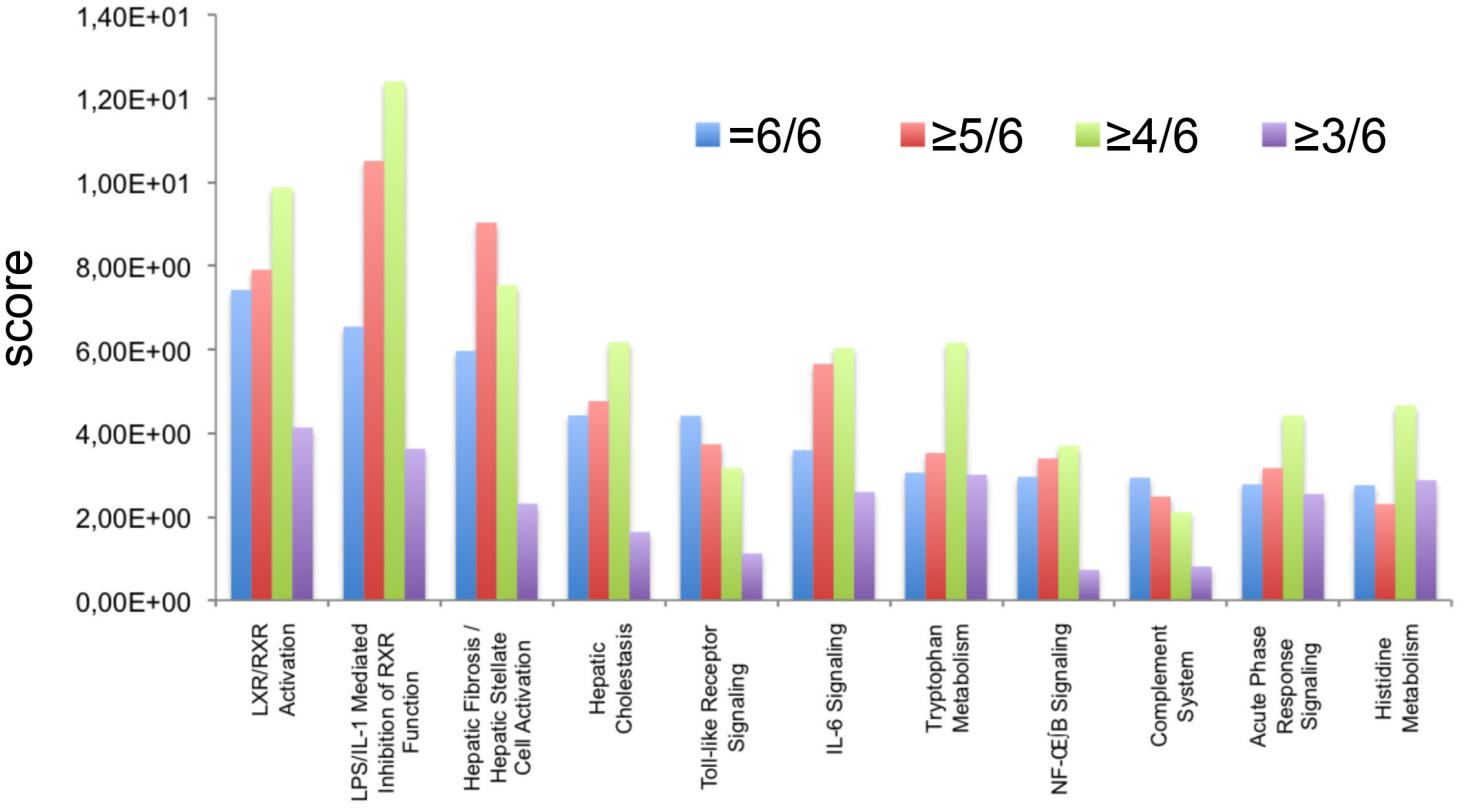
Comparative analysis of the most significant Canonical Pathways throughout the entire dataset, and across multiple datasets. The first 10 canonical Pathways generating significant scores are displayed as a bar chart along the x-axis. The y-axis represents the IPA score: the taller the bar, the better the score for the indicated pathway. For each canonical pathway, we have compared the progression of this EV value for increasingly tolerant values by decreasing EV occurrence;  = 6/6: blue; ≥5/6: red; ≥4/6: green; ≥3/6: violet.

Consequently, an occurrence rate of ≥4/6 gave us the most comprehensive and relevant information on the differentially expressed genes associated with the LPS activation. We observed that the number of genes associated to the 10 best canonical pathways (Figure 1) increases in most (7/10) of the IPA identified pathways until an occurrence rate of 4/6, but drops at an occurrence rate of 3/6. This observation strengthened our conviction that the EV≥4 occurrence threshold was the optimal setting. Taken together, these data show that by decreasing EV occurrence, the number of genes considered to undergo expression variation can be increased for the analysis of macrophage activation by LPS. If relevant, the newly selected genes structurize and interconnect the networks to reach a maximum value for an EV occurrence≥4/6. At less stringent EV occurrence values (≥3/6), the stronger networks lose their structure, and their organization is re-examined under the influence of less relevant genes. Therefore, we chose the EV value≥1.3 and the EV occurrence rate≥4/6. These parameters were used to select 300 genes, which have to be analysed.

### Analysis of the macrophage response to LPS at an EV occurrence≥4/6

**Figure 2:**
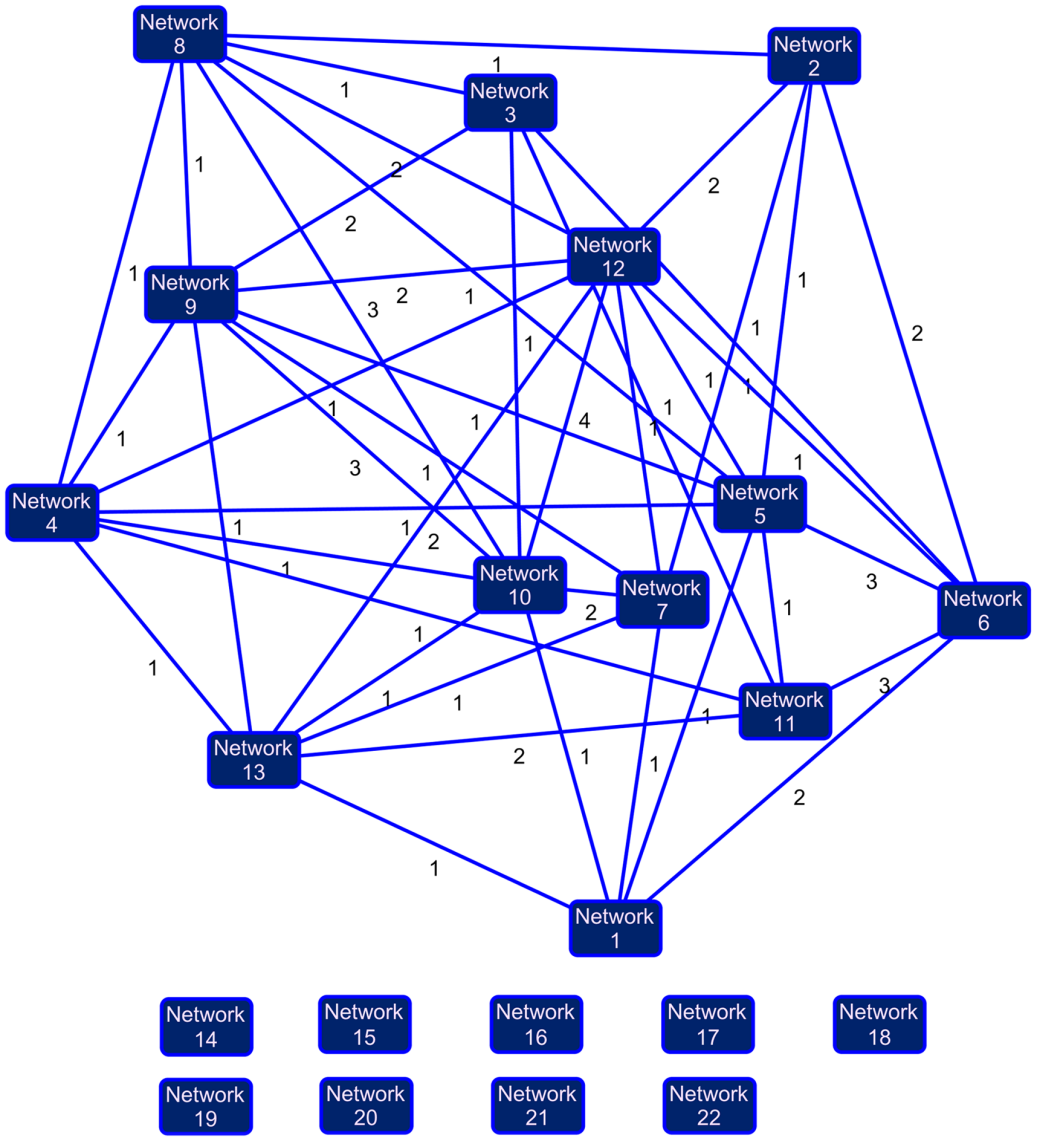
Interconnections between different networks. From our 195 differentially expressed genes, and the applied parameters (EV = 1.28; EV occurrence≥4/6), the data base has identified 22 different networks. The first 13 networks are heavily inter-connected as shown by solid lines between the networks. The integer beside each line indicates the number of genes that two networks have in common. Networks from 14 to 22 do not share common genes.

Most differentially expressed genes are grouped within a single meta network (Figure 2). Using EV threshold≥1.28 and EV occurrence≥4/6, three hundred genes having undergone significant expression variation were identified in our experiments. IPA mapped 277 genes, of which 202 could be associated to 22 networks. Thirteen of these are main networks interconnected by at least one common gene, and together form a meta network. The remaining 9 networks are deemed to be independent. For example, the major network #1 shares: a common gene product with network 5, two gene products with network 6, and one with networks 7, 10 and 13, respectively. The composition of each network is given in Table 3, which are classified into major and minor networks according to their score and to the number of genes identified and linked to these networks. The first twelve networks were identified as major networks with fairly high scores ranging from 38 for the best of them to 21 for the 12th. These identified networks are made up of gene products selected according to their EV ratio; varying from 23/35 (66%) for the first network to 15/35 (43%) for the 12th network. It is worth noting that the 13th network shares at least one common gene product with 7 of the major networks. Although it generated a low score (Score = 3), this network strongly overlaps with the other networks and can be considered as a member of the meta network presented in Figure 2. Consequently, the latter is deemed to be made up 13 major networks.

**Table 3:** Complete listing of genes within each network.

ID	Molecules in Network	Score	Focus Molecules	Top Functions
1	**ALDH7A1**, Angiotensin II receptor type 1, **CCL2**, CD3, **CD28**, **CD52**, Fabp, **FABP3**, **FABP5**, Fibrin, **FUCA1**, **GDF15**, **GNA15**, **ICAM1**, Ifn gamma, IKK, IL1, **IL18**, **LTA4H**, **Mmp**, MMP7, **MT1E**, NFkB, **PDPN**, peptidase, **PGDS**, **S100A4**, **SERPINA1**, **SGK**, **SLC2A6**, Sod, **SOD2**, **SPHK1**, TCR, **TIMP1**	38	23	Cancer, Cellular Movement, Hematological System Development and Function
2	**ABCE1**, **ACAT1**, **ANPEP**, Ap1, **AQP9**, **CDC42EP5**, F Actin, **FOXC2**, **FPR1**, Histone h3, **IFITM1**, Ige, Insulin, LDL, **LEP**, Mapk, **MCL1**, **MT1G**, **ND4**, **NMB**, P38 MAPK, **PBEF1**, Pkc(s), Pld, **PPARG**, **RGS2**, **RPS6KA1**, Rxr, **S100A8**, **S100A9**, **SCARB1**, **SHMT2**, **SNCA**, **UGP2**, Vegf	38	23	Lipid Metabolism, Molecular Transport, Small Molecule Biochemistry
3	**ADCY3**, Adenylate Cyclase, Alcohol group acceptor phosphotransferase, Alkaline Phosphatase, **APBB1**, **ATF3**, C13ORF15, Calmodulin, **CCL7**, **CCNB1**, Ccnb1-Cdc2, CCNB1IP1, **CD9**, **CD163**, **CDC2**, Ck2, **CKS1B**, Cyclin B, **CYP1B1**, E2f, **FCGR2A**, **HIST2H4A**, **LAIR1**, **LILRB2**, **MT2A**, **PBX3**, **PCSK1N**, **PILRA**, Pka, PLC, **PTPN6**, RNA polymerase II, **RRM2**, **STMN1**, **YBX1**	36	22	Cellular Compromise, Cell Death, Cellular Assembly and Organization
4	**A2M**, Akt, Calpain, **CCL13**, **CD14**, **CSF1R**, **CSF2RA** (includes EG:1438), **EIF4EBP1**, ERK1/2,**FGFR4**, Hsp70, **IER3**, **IL8**, **IL7R**, **ISG15**, Jnk, **MBP**, **NFKBIA**, **PAK1**, Pdgf, PDGF BB, PI3K, PLC gamma, PP2A, Rac, **RBP1**, STAT, STAT5a/b, Syk, Tgf beta, **TGFBI**, **TLR2**, **TNFAIP3**, **TREM1**, VAV	29	19	Hematological Disease, Organismal Injury and Abnormalities, Inflammatory Disease
5	**APOC1**, ARTS-1, **CCL18**, **CECR1**, CETP, DNMT3A, **EBP**, **EFNA3**, ethanol, GABPB2, GAPDH (includes EG:14433), **H2AFY**, HNRPA2B1, **IFI27**, **IFITM1**, IFNG, IL15, IL32, IL18RAP, **IL1R2**, IL1RAP, **IL7R**, INS1, ISGF3G, **KCNJ5**, KIR2DL3, **LGALS3BP**, **MNDA**, NKX2-1, **OAS3** (includes EG:4940), **PLTP**, **PRC1**, **SOD2**, **TAZ**, YY1	27	18	Lipid Metabolism, Molecular Transport, Small Molecule Biochemistry
6	ANXA1, **BPHL**, CD19, CD74, **EMP1**, FSTL1, GAL, GH1, **GPNMB**, GRB2, **HADH**, IL6, IL1RAP, ISGF3G, **KIF22**, **LGMN**, **LY9**, **MICAL1**, **MT1E**, MYC, NTS (includes EG:4922), PNPT1, PPP1R15A (includes EG:23645), **RNASE6**, **RRM2**, **SCARB1**, **SEMA4A**, **SHMT2**, **SNX10**, **SOD2**, TAF9, TAL1, TNFRSF10B, **TTC3**, UBE2C	25	17	Cellular Growth and Proliferation, Metabolic Disease, Immune Response
7	**ALDH1A1**, Angiotensin II receptor type 1, **BTG1**, CCL20, CD38, **CMTM3**, DCN, **DHRS9**, EDNRA, ELAVL2, HOXA5, HOXA9, HOXB9, **HS3ST1**, **HS3ST2**, **IER2**, IGFBP7, **IMPDH2**, KRT5, **KRT14** (includes EG:3861), MAPK1, MAPK12, **PNRC1**, PRMT1, retinoic acid, **RPL7**, **S100A8**, **SERINC2**, SERPINB9, **SLA**, **SLC5A3**, SOX4, sulfotransferase, TSC22D3, **ZFAND6**	23	16	Cellular Growth and Proliferation, Cancer, Cellular Development
8	ACP5, **ALOX5AP**, **C5ORF13**, CALCR, **CCL7**, **CCL18**, **CD14**, **CDCA7L**, CEBPE, **CHST2**, CSN2, FCER2, **FPR1**, Hsp27, IL9, IL13, IL15RA, **IL4I1**, MAO, **MAOA**, Metalloprotease, **MT1X**, **PLCXD1**, **PPA1**, PTGER2, PTGER4, PTGS2, **QSOX1**, **SAMSN1**, **TM7SF4**, TNFRSF11A, TNFRSF11B, TNFRSF1B, TNFSF11, Ubiquitin	23	16	Embryonic Development, Tissue Development, Tissue Morphology
9	BDKRB1, C1q, **C1QA**, **C1QB**, C1R, **CHST2**, CIDEC, **CKS1B**, CR1, **EEF1B2**, ERBB2, **FDFT1**, **ISG15**, ITM2B, **KIFC3**, LCN2, **LGALS3BP**, MHC Class II, **MT2A**, NID1, **NINJ1**, **PABPC4**, PGM1, PTEN, **RAB34**, **SEC61A1**, **SNN**, SOX4, TFAP2C, TNF, TNFRSF9, **TPST1**, TPT1, UCK1, WAP	23	16	Hepatic System Disease, Liver Hepatomegaly, Cell Signaling
10	**ADAMDEC1**, Angiotensin II receptor type 1, beta-estradiol, **CKS1B**, DDIT4, FMO5, GABRB2, **GCHFR**, **GM2A**, **GRINA**, **HIST2H2AA3**, HIST2H2BE, hydrogen peroxide, **IFI6**, IFNB1, **ISG15**, LCN2, **LILRA2**, **MAOA**, MPZ, MSGN1, NPY1R, PDZK1IP1, PIGR, **PMP22**, **PNRC1**, POMC, PPBP, progesterone, PTGER2, PTGER4, SLC3A2, **SLC7A11**, **TBX19**, **TRAK2**	21	15	Neurological Disease, Cellular Growth and Proliferation, Amino Acid Metabolism
11	**ACE**, BBC3, CASP3, Caspase, CSNK1D, CTSD, EIF4B, **FLJ11259**, **GLIPR1**, **HIST1H2AD**, HMGB1 (includes EG:25459), HNRPU, **HOXC6**, **IER3**, **IFITM2**, **IFITM3**, IGFBP3, **IKIP**, IRF5, ISGF3G, **KIAA0101**, MED21, NFYB, **PER3**, **RRM2**, **SLC39A8**, SMARCA4, SNCAIP (includes EG:9627), TAF9, TBP, TFAM, **TFB2M**, **TNNI2**, TP53, UBE2D3	21	15	Cancer, Cell Death, Gastrointestinal Disease
12	**ALOX5AP**, CD58, **CDC42EP3**, CEBPB, CNR2, cyclic AMP, FAAH, **GATM**, **GCSH**, glycine, **HLA-DPA1**, HLA-DPB1, HP, IGHE, IL4, IL1B, IL1RAP, **ISG15**, LCN2, **MARCO**, Mhc2 Alpha, **NPW**, ORM2, PIGR, PTGER4, **S100A8**, **S100A9**, SCGB3A1, **SEMA4D**, **SLC2A9**, SPRR1A (includes EG:6698), TGTP, **TMEM176A**, **TMEM176B**, **TREM2**	21	15	Immune Response, Cellular Movement, Hematological System Development and Function
13	adenosine, **AHCY**, AR, ATM, ATP, CASP3, E2F1, **EIF2S3**, **FAIM**, GZMB, HGF, hydrogen peroxide, IL2, IL4, IL1B, Jnk, NAD+, NFkB, NFKB1, nitric oxide, NMNAT1, P2RX7, PARP1, PCNA, PRKCD, PRKCE, PRKDC, retinoic acid, **SRGN**, TNF, TP53, XRCC5, XRCC6, YWHAZ, ZIC2	3	4	Cell Death, Hematological Disease, Immunological Disease
14	**ACAD9**, Acyl-CoA dehydrogenase	2	1	Amino Acid Metabolism, Carbohydrate Metabolism, Lipid Metabolism
15	NSD1, **ZNF496**	2	1	Developmental Disorder, Genetic Disorder, Embryonic Development
16	Malate dehydrogenase (oxaloacetate-decarboxylating) (NADP), **ME3**	2	1	Energy Production, Free Radical Scavenging, Cellular Function and Maintenance
17	Aldose 1-epimerase, **GALM**	2	1	Carbohydrate Metabolism
18	**ARSK**, Aryl Sulfatase	2	1	Lipid Metabolism
19	DIRAS3, **MT1B**	2	1	Cellular Development, Cancer, Cellular Growth and Proliferation
20	**CRTAP**, PSCD2, PSCD3	2	1	Cell Morphology, Cellular Assembly and Organization, Cell Signaling
21	DPEP, **DPEP2**, leukotriene D4	2	1	Cell Signaling, Immune Response, Cellular Assembly and Organization
22	**SMS**, Spermidine synthase, Spermine synthase	2	1	Amino Acid Metabolism, Small Molecule Biochemistry, Developmental Disorder

The genes found to be differentially regulated in our experiments and the number of such genes displayed in the “Focus molecules” column have been highlighted in bold print. The score is generated using a p-value calculation and is displayed as the negative log of that p-value. This score indicates the likelihood that the assembly of a set of focus genes in a network could be explained by random chance alone. A score of 2 indicates that there is a 1 in 100 chance that the focus genes are together in a network due to random chance. Therefore, networks with scores of 2 or higher have at least a 99% confidence of not being generated by random chance alone. The data base attributed general cellular functions to each network which are determined by interrogating the Ingenuity Pathway Knowledge base for relationships between the genes in the network and the cellular functions they impact.

**Figure 3:**
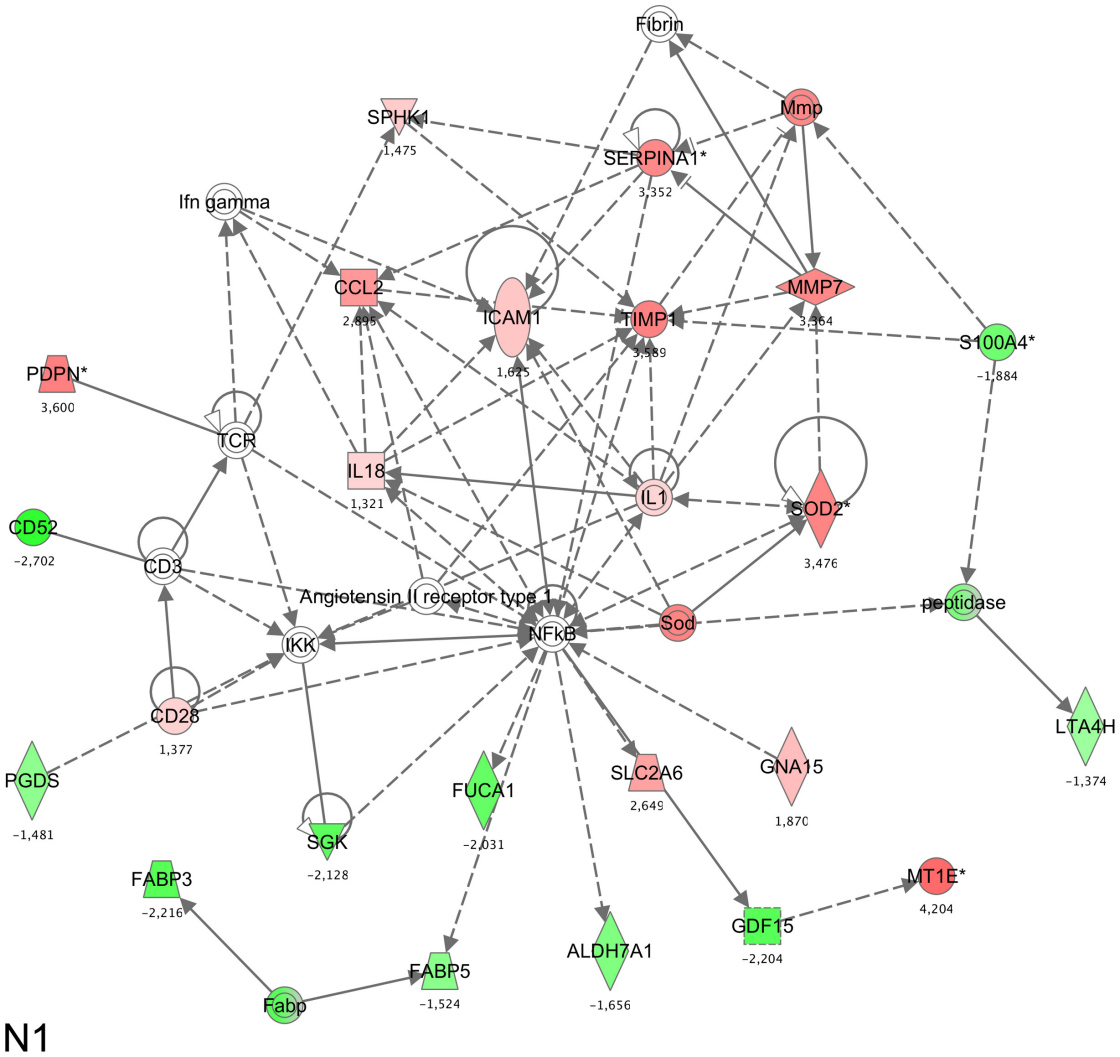
Close up of network. A maximum authorized number of 35 genes were used to generate a network. Direct interactions between each gene within a network were represented. Genes highlighted in green were down-regulated whereas genes in red were up-regulated. The number beside a gene name indicates its fold change expression. Genes in white, which were not found in the assay, were added by the data base as they are relevant to the network. Solid lines represent a direct interaction whereas a dashed line represents an indirect interaction.

The other secondary networks are comprised of few gene products and cannot be directly associated to major networks, which signifies that only 9 out of the total of 202 gene products were not associated to major networks. In Figure 3, we present in more detail, the most likely network (i.e. network 1). It contains 10 under-expressed genes, which products are coloured green, and 13 over-expressed genes, which products are coloured red. The others molecules were not detected as variant in our experiment. This principal network is centred on NF-*kappa*B, which is known to play a central role in LPS activation. Figure 3 also shows that NF-*kappa*B interacts and upregulates target genes as illustrated by ICAM1.

**Figure 4:**
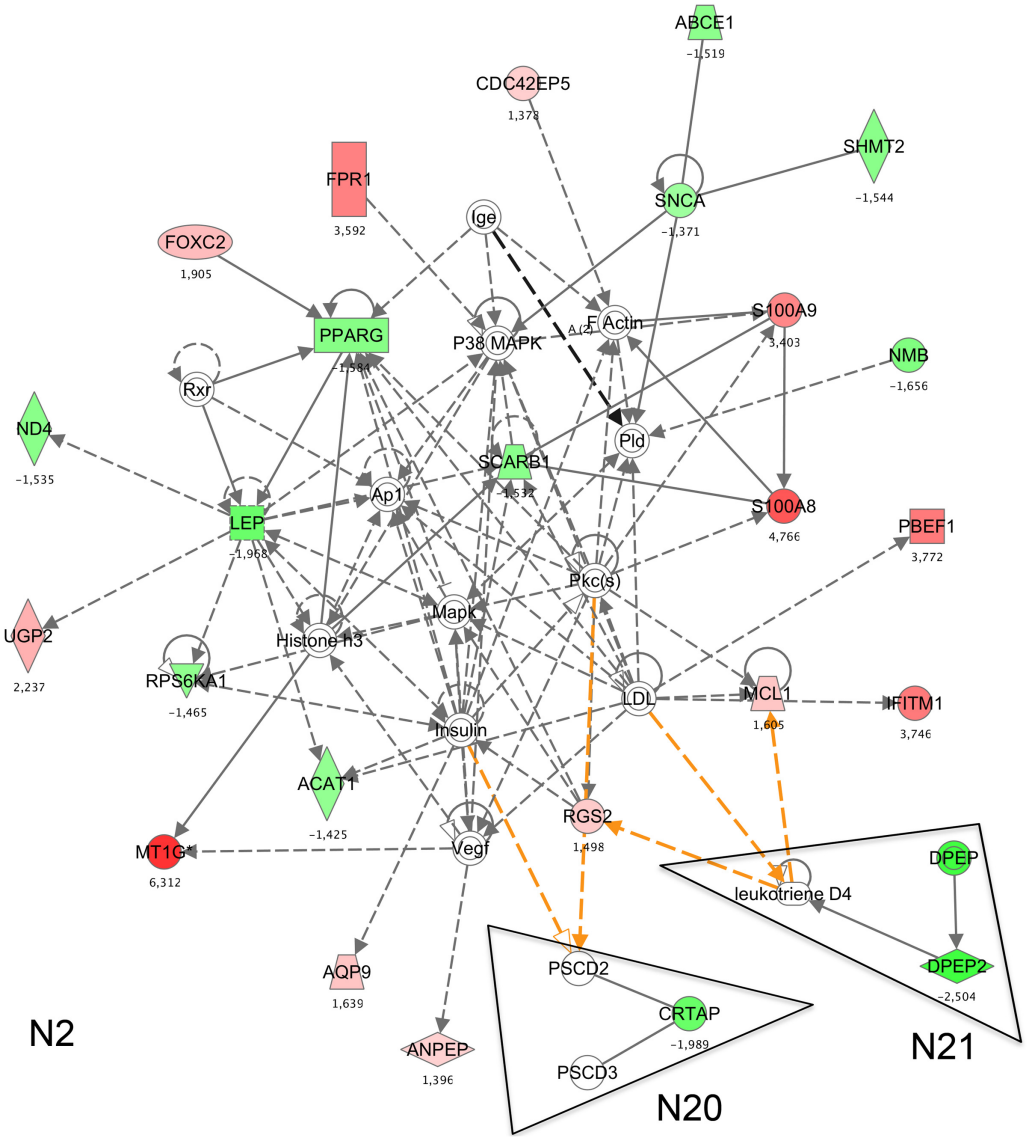
Connection of network 2 with minor networks. Networks are built as previously described in Figure 3. Genes that are in green were down-regulated whereas genes in red were up-regulated. The number beside a gene name indicates its fold change expression. Genes in white, which were not found in the assay, were added by the data base as they are relevant to the network. Solid lines represent direct interaction between gene products whereas dashed lines represents indirect interaction. Orange lines display interconnections between minor networks (N 20 and N 21) and major network 2.

At this level of analysis, the minor networks, which were at first considered to be less pertinent, should be re-analysed. For instance, network 21 structured around DPEP2 (dipeptidase 2) interacts through leukotriene D4 with up to three gene products belonging to 9 of the main networks. Similarly for network 20, CRTPA interacts through PSCD gene products with five main networks (data not shown). The interconnection of network 20 and 21 with the main network 2 is given in Figure 4. Overall, among the 202 eligible gene products from a knowledge database, 193 gene products were highly interconnected through 63 common gene products. The above genes structure the previously mentioned Meta network (Figure 2).

#### Genes and molecular pathways affected by LPS stimulation

Among the 300 macrophage genes undergoing expression variation upon LPS stimulation, the pathway analysis knowledge database has revealed that 178 gene products could be defined as “Top Bio Functions” thanks to their score (data not shown). Most relevant functions were associated with cell signalling (85 gene products; p = 2.14×10^−47^), cancer (62 gene products; p = 1.17×10^−40^), cell death (65 gene products; p = 9.07×10^−39^), cell growth and proliferation (53 gene products; p = 8.29×10^−30^), immune response (35 gene products; p = 4.87×10^−29^), inflammatory diseases (40 gene products; p = 1.51×10^−27^) and immune diseases (30 gene products; p = 4.63×10^−25^). These are strongly overlapping bio functions and a number of differentially expressed genes were common to two or more categories. Therefore, almost 60% of our selected genes were closely linked to cellular responses and/or cellular activation processes.

**Figure 5:**
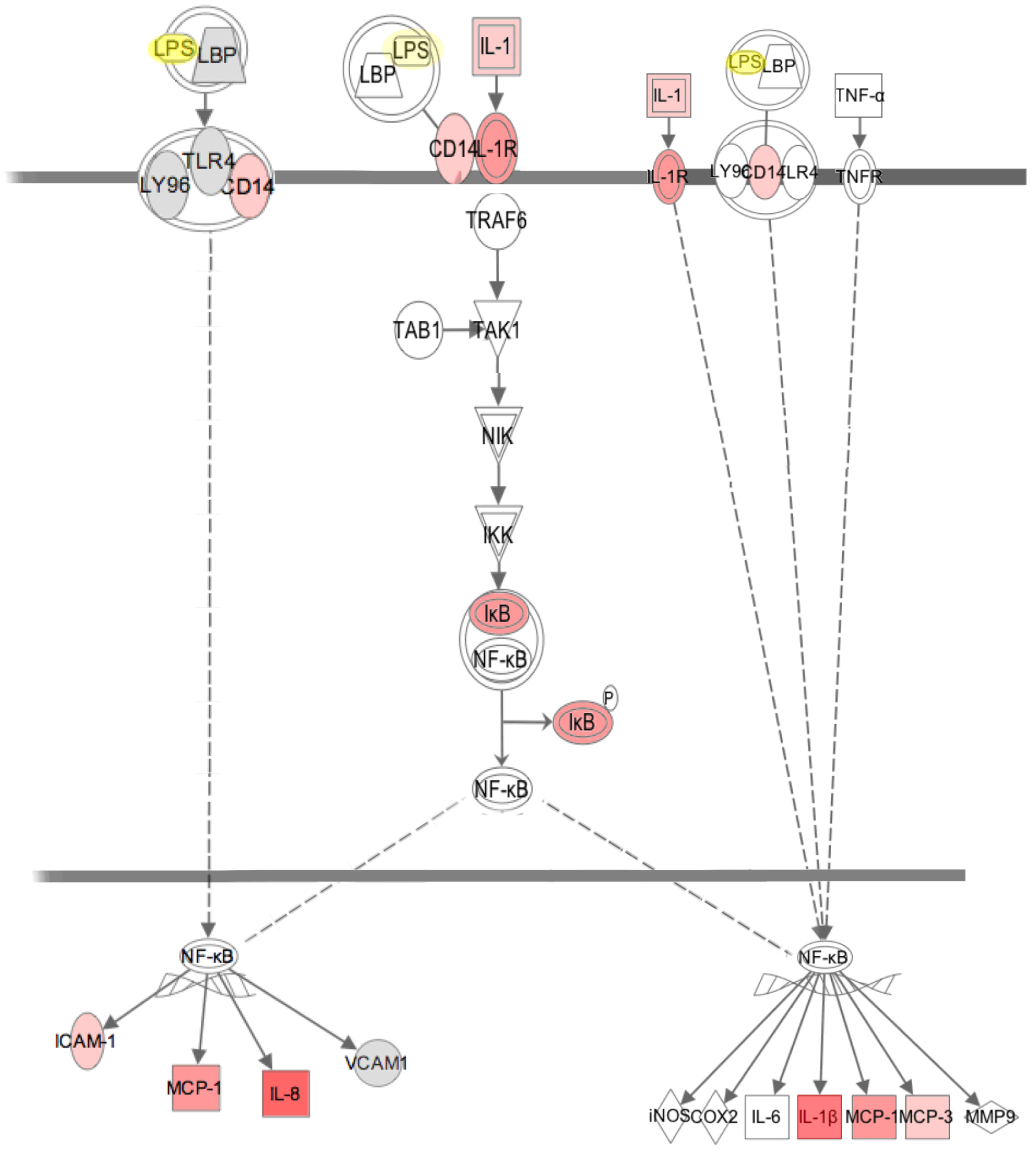
Canonical pathway of differentially regulated genes after LPS stimulation mediated by the NF-*kappa*B pathway. Graphical representation of the metabolic pathway LXR/RXR activation exhibited as the main metabolic pathway by the data base according to the best EV value selection. The Toll-like receptor signalling pathway enables the production of cytokines with activation of NF-*kappa*B.

CD14 and NF-*kappa*B target genes such as IL 1-/3, IL8, ICAM1, MCP1, or MCP3 were up regulated upon LPS stimulation of macrophages. These regulated genes are displayed in the canonical pathways (Figure 5), and results are consistent with LPS macrophage activation. It is worth noting that no expression variation was observed for the COX-2 or i-NOS2 genes involved in the generation of the oxidative burst. Another interesting finding was that a number of genes undergoing expression variation after LPS stimulation were involved in cellular lipid metabolism (Figure 2B). These include: the PPARγ nuclear receptor, genes coding proteins involved in fatty acid and cholesterol transport (FABP3, FABP5, SCARB1) or enzymes involved in cholesterol or lipid metabolism (ACAT1). Accordingly, LPS/IL-1 mediated inhibition of the retinoid X receptor (RXR) function and liver X receptor (LXR)/RXR activation was found to be the most significantly affected pathway after 48 hours of LPS stimulation (Figure 1). Finally, and most surprisingly, six metallothionein genes are up regulated and found among the networks (MT2A in network (N) 3 and N9, MT1B in N19, MT1E in N1 and 6, MT1F in N2, MT1G in N2, MT1X in N8). One pseudogene (C20ORF127 metallothionein pseudogene 3) was also up regulated.

### Estimation of inter-individual variability

After having verified intra chip homogeneity we evaluated inter individual variability of the transcriptomic response. For each individual, the set of genes with EV≥2.32 (p≤0.01) were selected and compared two at a time. Maximum gene overlapping between donors ranges between 43.9% and 77.6% (data not shown). For paired individuals, all differentially expressed genes shared an average overlap of 54%. We can therefore estimate the variability level between two donors to be 46%. From this value it was impossible to discriminate between biological and technical variability. Nevertheless, technical variability has been evaluated in reproducibility studies [21] and estimated to average 19.5%. For our experiment, we considered technical variability to be 20%. This calculation left us with a 26% average variability rate due to individual (biological) factors. Using these (individual+technical) variability estimations we calculated the number of expected genes according to an EV occurrence. According to our observations 300 genes are differentially regulated at an EV≥4/6. Given an average inter individual variability of 46%, we expect to obtain 162 differentially expressed genes at an EV occurrence≥5/6 and 87 differentially expressed genes at an EV occurrence = 6/6.

## Discussion

In recent years, micro-array studies have been increasingly used to analyse tissue or cell response to a given stimuli [3], [22]–[25], and provide a lot of data for analysis. However, improved integration of this huge mass of data is needed to better understand the biological processes for which slight modifications in gene expression can have significant consequences. In addition, study design and/or statistical types of analysis only allow the detection of genes undergoing the most significant expression variations, and can only be seen as semi-quantative methods [26] at best. Indeed, these studies may miss important genes which undergo slight expression variations or appear as such on the micro-arrays. To overcome this problem we describe an approach that we have named “Expression Variation Occurrence Analysis”. We linked this approach to biological parameters to improve their threshold parameter so that settings could be optimized. We then applied our approach to the LPS-activated macrophages model to experimentally verify and validate our prediction on a well-known activation pathway.

Because of the growing quantity of data contained in databases, we chose software, which is powerful enough to analyse the largest number of genes within the same network. This was done to illustrate the robustness of our analysis.

We have observed that by fine-tuning the stringency level setting, some of the parameters simultaneously reached *extremum* values, which indicated that optimum setting had been obtained. Furthermore, at this threshold the transcriptional signal is highly structured within a meta-network with a large number of genes interconnecting major and minor networks. This structuring effect is lost at lower EV occurrence thresholds, indicating that the genes selected are not relevant to LPS macrophage activation, which concurs with current knowledge. In our study only 3% of the 300 genes were estimated to be nonsignificant whereas in another study using MPSS measurements more than 20% of 127 genes were deemed to be nonsignificant [27].

The optimisation of the EV threshold/occurrence rate to analyse the differentially regulated gene allowed us to confirm the existence of a macrophage activation process mediated by the TLR4/NF-*kappa*B pathway [28]–[30]. CD14, which interacts with LBP to present LPS to TLR4 [31], was also found to be up regulated [32] as well as a number of genes known to be NF-*kappa*B targets or that have putative KB sites. Among those genes, some promoted the inflammatory response (IL8 [33]–[34], MCP1 [35], MCP3 or ICAM1 [36]). Other genes such as NF-*kappa*B or SOD2 (encoding a free radical scavenging enzyme), played a key role in reducing the extent the oxidative burst and hence cell damage [37]. We were able to predict that some of the main up regulation activity was not detected such as with NOS2 or COX2, which encode for the main enzymes involved in reactive nitrogen and oxygen species (RNS and ROS) production upon LPS activation of macrophages. Because of late RNA extraction (48 hours after LPS stimulation) we did not expect to see differentially expressed gene that display an early and transient response during the macrophage activation process [38]. Thus our results are in agreement with those found for late extraction, in the literature. A last significant finding was that an important subset of identified regulated genes are involved in Lipid transport and metabolism, which concurs with the fact that nuclear receptor (LXR and RXRs) pathways were identified by automatic analysis and that the peroxisome proliferator-activated receptor γ (PPARγ) gene was found to be down regulated. PPARs and LXRs are transcription factors activated by the products of lipid metabolism [39], and are involved in regulating lipid metabolism and cellular cholesterol homeostasis. Recent findings have also shown that they play an important role as negative regulators (in association with RXR receptors) of macrophage-mediated inflammation [40] presumably through a mechanism of trans-repression directed mainly at transcription factors such as NF-*kappa*B and AP-1 [41]. Analysis of differentially expressed genes with an appropriate EV occurrence would appear to adequately reflect the macrophage response to LPS activation and has highlighted the fact that mechanism of macrophage inflammation regulation may be triggered in the late phase of macrophage activation. This is illustrated by evidencing overexpression of metallothionein (MT) genes, which is known to be induced by inflammatory stress [42], [43]. In addition, MT genes carry specifically adapted functions, which are tightly regulated through their expression as discrete isoforms [44].

Interestingly a large proportion of genes involved in lipid metabolism, such as PPRγ, ACAT1, SCARB1 [45], FABP5 [46], FOXC2 [47] were only found to be differentially expressed with a more stringent EV occurrence rate and would not have been detected by the approach where mean EV was calculated for all the 6 donors. Other genes modulating the inflammatory response, such as the MARCO scavenger receptor [48], IKKP (an inhibitor of NFκBIA) or the macrophage inhibitory cytokine (MIC [49] or GDF15) fall into the same category. This illustrates the sensitivity and power of the EV occurrence analysis strategy when used to describe cell transcriptomic response and to highlight the subtle nature of regulation mechanisms. In addition to identifying genes with low EV values and robust statistical evidences (p = 10^−6^ for a gene with EV≥1.28 in all donors), EV occurrence analysis provides an additional framework for the analysis of differentially regulated genes. Genes commonly regulated in all donors can be distinguished from those for which expression is associated with inter-individual expression heterogeneity and only detected at lower occurrence values. Genes found to be over or under expressed in all individuals can be seen as important genes involved in the response to the condition under study. This is illustrated by NF-*kappa*B target genes or Metallothionein genes in our study. Those evidenced at lower EV occurrences, as in the case for a subset of lipid metabolism genes, may be genes with minor expression differences and/or displaying differences in expression behaviour between individuals. This is an important point to consider in the context of the study of genetic susceptibility to complex diseases. This is particularly true, as the proposed workflow can be used to highlight differentially expressed genes of interest that would not have been included in a standard analysis because they exhibit inter-individual variability for the same stimulus. Indeed, genes displaying inter-individual expression heterogeneities may be seen as candidate genes that highlight the genetic variability of individuals.

To conclude, we consider that EV occurrence analysis may be a useful tool for analysing human cell behaviour in reaction to unknown stimulus (such as cancer or pathogens). Differential gene expression can thus be detected using robust statistical evidence, even for genes with low expression differences, and the method described above provides us with a more complete picture of the transcriptomic response. Furthermore, it has the ability to identify inter-individual differences in the cellular response that can be linked to disease susceptibility.

## Materials and Methods

### Cell Preparation and Culture Conditions

This study was conducted according to the principles expressed in the Declaration of Helsinki. The study was approved by the Institutional Review Board of l'Etablissement Français du Sang Aquitaine Limousin (place Amélie Raba Léon - 33000 Bordeaux - FRANCE), and l'Université Victor Segalen - Bordeaux II (Laboratoire de Parasitologie, led by Professor Philippe VINCENDEAU; 146 rue Léo Saignat - 33000 Bordeaux - FRANCE, (ethics approval réf. CPIS 10.11). All healthy volunteers provided written informed consent for the collection of samples and subsequent analysis. Human monocytes were obtained were free of any infection and with no history of medical therapy in the previous two weeks. All samples were processed together the same day. Briefly, the mononuclear cell fraction was obtained by gradient centrifugation over Ficoll (Histopaque 1077, Sigma-Aldrich Chimie, Lyon, France) and monocytes were purified by CD14+ magnetic cell sorting (Miltenyi Biotech) according to manufacturer's instructions. Monocytes were then seeded in 24-well plates at 10^6^ cells per well (4 wells per donor) in complete Iscove's Modified Dulbecco's Medium (IMDM) supplemented with 10% heat-inactivated human type AB (Cambrex) serum, ultra-glutamine 2 mM, penicillin 200 U/ml and streptomycin 200 pg/ml. Cells were cultured for 5 days (with pre-warmed complete medium change at day 3) to allow differentiation into macrophages. At day 5, two wells per donor were stimulated with 100 ng/ml lipopolysaccharide (LPS, *Escherichia coli*, Sigma) whereas the two other wells were left as unstimulated controls (medium change only). After 48 hours, all wells were washed with pre-warmed phosphate-buffered saline (PBS). Total RNA was then extracted from the freshly pooled monolayer in duplicate experiments, which includes a DNAse step, using the RNeasy® mini kit (Qiagen) according to the manufacturer's instructions. RNA quantity and purity was quantified using a NanoDrop ND-1000 spectrophotometer (Nanodrop Technologies) and assayed for degradation using an Agilent bioanalyser (Agilent technologies, Santa Clara, USA) according to the manufacturer's instructions.

### RNA labeling, hybridization and Microarray data acquisition

One microgramme of each RNA was used to generate either Cy3- or Cy5- RNA amplification (aRNA) target [50] using the Amino Allyl Message Amp II aRNA amplification kit (Ambion, Applied Biosystems, Courtaboeuf, France), according to manufacturer's instructions. The macrophage transcriptional profile of the 6 donors (being stimulated or not, by LPS) was evaluated independently using the Operon Human Genome Array-Ready Oligo Set™ (AROS, Operon Technologies) Version 4.0, containing 35,035 oligonucleotide probes representing approximately 25,100 unique genes and 39,600 transcripts. Prior to hybridization, excess oligonucleotide was removed from the arrays by shaking them twice for 1 min in 0.2% SDS. Arrays were then washed twice in distilled water. The two labelled aRNA were added to version 2 of microarray hybridization buffer (GE Healthcare) in a final 50% formamide concentration, denaturated at 95°C for 3 min and applied to the microarrays in individual chambers of an automated slide processor (GE Healthcare). Hybridization was carried out at 37°C for 12 hours. Hybridized slides were washed at 37°C successively with 1×SSC, 0.2% SDS for 10 min, twice with 0.1×SSC, 0.2% SDS for 10 min, with 0.1×SSC for 1 min and with isopropanol before air drying. Microarrays were immediately scanned at 10 µm resolution in both Cy3 and Cy5 channels with a GenePix 4200AL scanner (Molecular Devices) with a variable PMT voltage to obtain maximal signal intensities with <0.1% probe saturation. ArrayVision software (Alpha Innotech, Santa Clara, USA) was used for feature extraction. Spots with high local background or contamination fluorescence were flagged manually. A local background was calculated for each spot as the median values of the fluorescence intensities of 4 squares surrounding the spot. This background was subtracted from the foreground of fluorescence intensity. All data is MIAME compliant, the raw and normalized data has been deposited in Gene Expression Omnibus (GEO) database (GSE22858).

### Measure of Expression variation and gene selection

Identification of differentially regulated genes was carried out using the EV (Expression Variation) method [21], on paired data, i.e. LPS stimulated macrophages versus unstimulated macrophages from the same donor. This method can be used to analyze the signal with regards to noise by normalizing and building confidence bands of gene expression, and by fitting cubic spline curves to the Box–Cox transformation. The confidence bands, fitted to the actual variance of the data, include the genes devoid of significant variation, and are used to calculate EV, based on the confidence bandwidth. Each outlier is positioned according to the dispersion space (DS) and provides a measure of gene EV associated with a p-value. This model allows us to stabilize variance.

Two approaches were then used to select genes for further analysis. Firstly, genes with a mean of p-value≤0.01 were considered to be differentially regulated for different macrophages culture conditions. Secondly, for each gene, we determined an EV occurrence parameter corresponding to the number of time the gene was found to vary in the same way among donors. In this case we set a low statistical threshold for EV values with (p≤0.1) for inclusion of genes in the analysis.

As the transcriptomic chips Operon© are embedded with probes targeting the same gene, we compared the homogeneity of EV values (EV≥1.28 EV occurrence≥4/6) of duplicated genes present on the array. The mean, the median and standard deviation of the replicates are equal to 7.35%, 6.15% and 5.88% respectively, indicating that EV measurements are robust and below 30% thus corresponding to an EV = 1.28.

### Pathway Analysis

The functional analysis algorithm developed by Ingenuity Pathway Analysis (IPA) (Ingenuity® Systems Inc., Redwoodcity CA. USA, http://www.ingenuity.com) was used to identify the networks, biological functions and/or diseases that were most relevant to the dataset (i. e. the selected differentially expressed genes). For this analysis, we selected the median value of expression levels (calculated for the 6 individuals) as the measurement of the level of expression considered for IPA. This enables, in the case of the occurrence method, the minimization of the extreme values effect and means that only the relevant measurements calculated for the selected individuals are retained.

#### Network Generation

A dataset containing gene symbols and the corresponding expression values was uploaded into the application. Each gene identifier was mapped to its corresponding gene object in the Ingenuity Pathways Knowledge Base. The genes identified as significantly differentially regulated by IPA, called focus genes, were overlaid onto a global molecular network developed from information contained in the Ingenuity Pathways Knowledge Base. Networks of these focus genes were then algorithmically generated, based on their connectivity. A network was limited to a maximum of 35 genes, which were associated according to their functional connections. We considered a network to be major if it was comprised of at least 20 differentially expressed genes. When necessary, some genes were added to complete the network structure in accordance with literature data.

#### Network Graphical Representation

A network is a graphic representation of the molecular relationships between genes or gene products. Genes or gene products are represented as nodes, and the biological relationship between two nodes is represented as an edge (line). All edges are supported by at least 1 reference from the literature, from a textbook, or from canonical information stored in the Ingenuity Pathways Knowledge Base. Human, mouse, and rat orthologs of a gene are stored as separate objects in this base, but are represented as a single node in the network. The intensity of the node colour indicates the degree of up- (red) or down- (green) regulation. Nodes are displayed using different shapes that represent the functional class of the gene product. Edges are displayed with different labels that describe the nature of the relationship between the nodes (e.g., P for phosphorylation, T for transcription).

#### Functional Analysis of a Network

The Functional Analysis of a network identifies the biological functions and/or diseases that are most significant to the genes in the network. Fischer's exact test was used to calculate a p-value determining the probability that each biological function and/or disease assigned to that dataset is due to chance alone. The network genes, associated with biological functions and/or diseases in the Ingenuity Pathways Knowledge Base, were considered for the analysis. Fischer's exact test was used to calculate a p-value which determined the probability that each biological function and/or disease assigned to that network is due to chance alone.
